# Feeding Practices Used by Australian Parents of Young Children Living With Food Insecurity and Household Chaos

**DOI:** 10.1111/mcn.13770

**Published:** 2024-11-25

**Authors:** Smita Nambiar, Lauren Stanley, Lily Miller, Rebecca A. Byrne, Danielle Gallegos, Robyn A. Penny, Kimberley A. Baxter

**Affiliations:** ^1^ School of Exercise and Nutrition Sciences, Faculty of Health Queensland University of Technology Kelvin Grove, Brisbane Queensland Australia; ^2^ Centre for Child Nutrition Research Faculty of Health, Queensland University of Technology South Brisbane Queensland Australia; ^3^ Child Health Liaison Children's Health Queensland Hospital and Health Service Brisbane Queensland Australia

**Keywords:** child, food insecurity, household chaos, responsive feeding

## Abstract

Responsive feeding practices are crucial for developing healthy eating behaviours in children. However, chaotic households and financial stress may disrupt these practices. This cross‐sectional study aimed to characterise feeding practices among Australian parents experiencing financial hardship. Parents of children aged 5–35 months, who identified as experiencing financial hardship, completed an online questionnaire from October 2021 to June 2022. Validated tools gathered data on feeding practices, mealtime structure and environment, household chaos (HC), household food insecurity (HFI) and sociodemographic characteristics. Bivariate correlations and hierarchical regression assessed relationships between these variables, adjusted for parent age, education and number of children. Data from 213 parent–child dyads were analysed (97% mothers, median age = 31 years, IQR 28–36; 50% boys, median age = 12 months, IQR 8–17). Median HC score was 4 (IQR 2–7). Seventy‐six percent of families reported experiencing HFI (median = 6, IQR 3–9). Over 80% of parents often or always ate meals as a family and never or rarely engaged in ‘parent‐led’ feeding (median = 1.75, IQR 1.00–2.50), or used ‘(non)‐food as reward’ (median = 1.33, IQR 1.00–2.00). ‘(Non)‐food as reward’ was positively correlated with HC (*p* = 0.016), and ‘food to calm’ was positively associated with HC (*p* = 0.004). ‘Feeding on demand’ was negatively associated with HC and HFI (*p* = 0.002). ‘Persuasive feeding’ was not associated with either. Findings suggest that HC had more influence than HFI on some nonresponsive feeding practices. Increasing levels of HC and HFI may result in less structured mealtimes. Interventions must consider how financial hardship, HFI and HC can impact parents' ability to engage in responsive feeding practices. This cross‐sectional study examined feeding practices among Australian parents facing financial hardship. Over 75% were food insecure. While the meal environment supported responsive feeding, increasing household chaos and food insecurity led to fewer structured mealtimes and household chaos increased coercive practices such as using (non)‐food rewards and food to calm.

## Summary

- Among families who self‐identified as experiencing financial hardship, three‐quarters were food insecure. A further 13% were marginally food secure.
- The family meal environment was conducive to responsive feeding. The use of coercive, nonresponsive feeding practices was low, yet increasing levels of HC and HFI reduced structured mealtimes.
- Household chaos appears to have a greater impact on the use of certain coercive practices including using ‘food to calm’ and using ‘(non)‐food rewards’ than household food insecurity.

## Graphical abstract

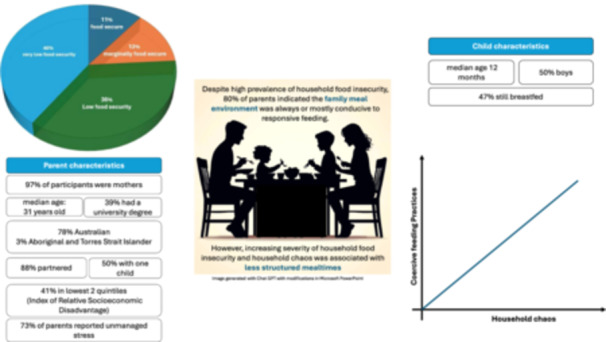

## 1 Background

Early childhood nutrition is a key determinant of health and wellbeing throughout life. Parents play a significant role in children's food intake. While it is important to consider *what* children are fed, it is becoming increasingly clear that *how* children are fed is equally important (Redsell et al. 2021). Feeding practices refer to the behaviours and strategies parents use to influence when, what and how much their child eats (Russell, Worsley, and Campbell 2015; Vaughn et al. 2015). The feeding practices implemented by parents in response to these cues are an integral part of parenting and can either support or hinder children's eating behaviour, food preferences, diet quality, growth, development and overall wellbeing (Aboud, Shafique, and Akhter 2009; Daniels 2019; Grammer et al. 2022; Harris et al. 2014; Russell, Worsley, and Campbell 2015). Feeding practices are influenced by parenting style and are based on two underlying dimensions of demandingness and responsiveness (Black and Aboud 2011; Habron and Booley 2013; Kuppens and Ceulemans 2019). Demandingness refers to the degree of control and supervision from parents, whereas responsiveness refers to warmth, involvement and sensitivity to a child's needs (Black and Aboud 2011; Habron and Booley 2013; Kuppens and Ceulemans 2019). These constructs have been developed with households that are relatively stable and free from trauma, including poverty.

Responsive caregiving is a fundamental component of nurturing early childhood development. The nurturing care framework defines responsive caregiving as observing and responding to children's movement and their verbal and non‐verbal requests, to protect children against injury, adversity and illness and foster learning, trust and social relationships (World Health Organization, United Nations Children's Fund, & World Bank Group 2018). In the context of feeding and food intake, responsiveness refers to reciprocity between parent and child, where parents provide safe, nutritious and age‐appropriate foods and the child decides how much to eat (Black and Aboud 2011; Hughes et al. 2005; Satter 1986). Parents learn to recognise and promptly respond to hunger and satiety cues from their child which preserves the child's ability to regulate their appetite and energy intake (Hughes and Frazier‐Wood 2016; Russell and Russell 2020). Parents also have an opportunity to model healthy eating behaviours and food preferences. Feeding occasions across the day present multiple opportunities for parenting and nurturing care (World Health Organization, United Nations Children's Fund, & World Bank Group 2018). The quality of nurturing care is highly important because consistent responsive parenting plays a unique role in a child's early brain development and has been shown to predict faster rates of cognitive and social growth (Landry, Smith, and Swank 2003; World Health Organization, United Nations Children's Fund, & World Bank Group 2018).

The concept map developed by Vaughn and colleagues (2015) proposed three overarching constructs and defined feeding practices and strategies within these constructs (Vaughn et al. 2015). The first domain, coercive control, is marked by low responsiveness and includes practices like restriction, pressure to eat, threats, bribes and using food to manage emotions. The second domain, structure, involves setting age‐appropriate rules and limits, such as clear expectations for mealtimes, guided choices, monitoring food intake, modelling healthy eating and ensuring food is well‐prepared and accessible. The last domain, autonomy support, describes child involvement in meal planning and preparation, encouraging children to eat healthy foods without consequence and using praise and negotiation. Autonomy support includes nutrition education, and combined with structure, is high in responsiveness and associated with lower levels of food fussiness, a higher intake of fruits and vegetables (Daniels et al. 2009; Daniels et al. 2015; Finnane et al. 2017; Magarey et al. 2016), normal growth trajectories (DiSantis et al. 2011; Paul et al. 2014; Redsell et al. 2021) and psychosocial development (Landry, Smith, and Swank 2003).

It is believed that responsive feeding may be contingent on stable environments with few distractions, so parents can adequately attend to the child during meals. Certain circumstances such as financial hardship may hinder a parents' ability to feed their child responsively (Arlinghaus and Laska 2021; Baxter et al. 2022). Families experiencing financial hardship may have unstable living arrangements and experience higher levels of household chaos (environmental confusion and disorganisation). High levels of chaos have been associated with poorer child development, reduced self‐regulation, reduced parental responsiveness and overweight/obesity (Fiese et al. 2016; Marsh, Dobson, and Maddison 2020; Saltzman et al. 2019). These families may also be food insecure. Food insecurity is defined as having ‘*a lack of regular access to enough safe and nutritious food an active and healthy life’* (Food and Agricultural Organization 2012). Depending on severity, food insecurity can impact the social, emotional, mental and physical wellbeing of adults and children (Gallegos et al. 2021).

In Australia, food insecurity is not regularly measured at the population level. Additionally, a variety of measurement tools can result in different prevalence estimates that may underestimate the problem (McKechnie et al. 2018). According to the 2015 Australian National Health Survey, food insecurity using the two‐item measure estimated that 4% of people ran out of food and could not afford to buy more, and a further 1.5% went without food when they could not buy any more. (Australian Bureau of Statistics 2015). Gatton and Gallegos (2023) placed the prevalence of moderate‐severe food insecurity (M‐SFI) in Australia at 8%–12% using the Food Insecurity Experience Scale (FIES) (Gatton and Gallegos 2023).

The impact of household food insecurity on parental feeding practices has not been extensively investigated in Australia (Arlinghaus and Laska 2021; Baxter et al. 2022; Redsell et al. 2021). This study aims to describe the feeding environment, mealtime structure and feeding practices of Australian families experiencing financial hardship. It will assess the prevalence of household food insecurity (HFI) in a group of parent–child dyads and investigate how the feeding environment, mealtime structure and practices are influenced by sociodemographic factors, HFI and household chaos (HC). The findings will be used to inform the development of an intervention to promote responsive feeding that will meet the needs of families experiencing financial hardship.

## 2 Methods

### 2.1 Study Design, Setting and Recruitment

This cross‐sectional study is part of a larger project called Responsive Feeding in Tough Times (RFiTT). RFiTT used mixed methods (codesign workshops, an online questionnaire, recorded observations of family mealtimes and interviews) to collaboratively design a responsive feeding intervention with families experiencing financial hardship across Australia (Baxter, Kerr, et al. 2024). The analyses presented here are based on data collected from the online questionnaire that were used to gain an understanding of the types of feeding strategies used by parents, which would then inform the content development of the intervention.

Parents across Australia were invited to participate if they had at least one child between 6 months and 2 years of age and answered ‘yes’ to the following screening question: ‘*Do you sometimes struggle to pay the bills?*’. The screening question was informed through consultation with parents to ensure language sensitively recruited individuals who were struggling financially and therefore at risk of food insecurity (Baxter, Kerr, et al. 2024). Due to limited research on the feeding practices used by families experiencing financial hardship, the aim was to recruit a convenience sample of at least 200 participants to describe the types of feeding practices used. This sample size would also allow for relationships between feeding practices and markers of financial hardship, such as household chaos and food insecurity to be investigated.

Recruitment was conducted from October 2021 to June 2022, which coincided with the latter stages of the COVID‐19 pandemic. Recruitment occurred through targeted paid advertisements on social media (Meta). Details of the study, in the form of electronic flyers, were also shared through relevant family, parent and budgeting‐focused pages on these platforms. Child health nurses and organisations that provided support and services to families assisted with promoting the study in their networks.

**Figure 1:**
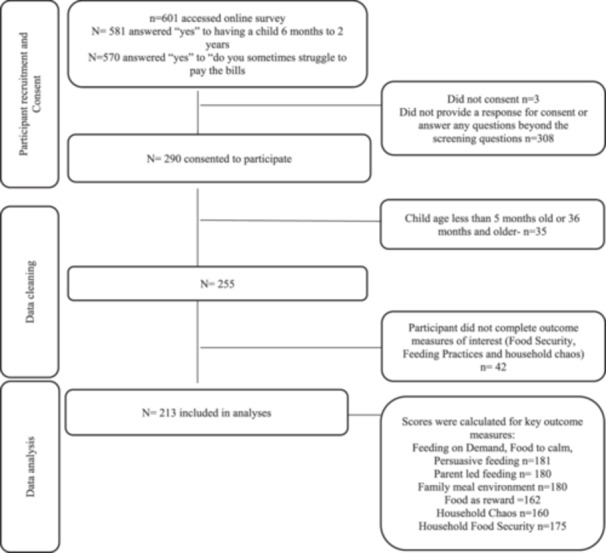
Participant flow diagram.

Following consent, participants completed the online questionnaire managed via the Research Electronic Data Capture (REDCap) tool hosted at QUT (Harris et al. 2019; Harris et al. 2009). If parents had more than one child within the specified age range, they were asked to complete the questionnaire for the child they had the most involvement in feeding. Participants were also invited to enter a competition for a chance to win one of four $50AUD gift vouchers for their contribution. This was not advertised in accordance with the human research ethics committee guidelines. Figure 1 describes participant recruitment, reasons for exclusion and final sample size.

### 2.2 Measures

The questionnaire included items on feeding practices, HFI, HC and sociodemographic information. Information on feeding practices was collected using the 34‐item Feeding Practice and Structure Questionnaire for Infants and Toddlers (FPSQ‐S) (Jansen et al. 2021). This questionnaire assessed the ‘family mealtime environment’ (4 items), ‘feeding on demand’ (4 items assessing mealtime structure) and four nonresponsive feeding practices: ‘using food to calm’ (6 items), ‘persuasive feeding’ (7 items) ‘parent‐led feeding’ (4 items) and ‘using (non)‐food rewards’ (9 items). All items in the FPSQ‐S had six response options on a Likert scale of never, rarely, sometimes, often, always and not applicable, coded 1–6. Two items associated with ‘feeding on demand’ and ‘parent‐led feeding’ were reverse‐coded as both these questions were child‐led, and all other items within these feeding practices were parent‐led. A score for each feeding practice was determined by finding the average of items. If a participant indicated ‘not applicable’, the response was not included in the calculations. Participants needed to provide a valid response to at least 75% of the items within a construct for a score to be calculated. For all practices, the higher the score, the greater the use of the practice. A higher score for ‘feeding on demand’ indicated less feeding on demand and more structure.

The 15‐item Confusion, Hubbub and Order Scale (CHAOS) was used to measure HC (Matheny et al. 1995). Participants responded true or false to each item, which was then scored as per Matheny et al. (1995), with a higher score indicating higher levels of environmental chaos (maximum possible score of 15) (Matheny et al. 1995).

The 18‐item United States Department of Agriculture Household Food Security Survey Module (USDA‐HFSSM) was used to assess HFI (Bickel et al. 2000). Following the guidelines in the survey module, participants were provided with four response options per question, which varied per question. Responses were then coded as negative, affirmative or missing. An imputation procedure was used to preserve data with missing responses. This procedure used the ordered character of items as described in the module. The order was considered applicable in the Australian context, where the severity of ordering items related to food insecurity tends to be stable across households (Seivwright, Callis, and Flatau 2020; So et al. 2024). From here, a score was determined by summation of the affirmative responses for households with children. Using the updated scoring guide (U.S. Household Food Security Survey Module: Three‐Stage Design, With Screeners 2012), a score of zero represented high food security; 1–2 represented marginal food security; 3–7 represented low food security and 8–18 represented very low food security. The scores could be further dichotomised, where high and marginal food security are classified as ‘food secure’ and low or very low food security are classified as ‘food insecure’.

Participants reported sociodemographic information: their age, height, weight, relationship status, relationship to child, number of adults in the household, number of children, type of housing, number of times they had moved to a new residence in the previous 12 months, ethnicity, highest level of education attained, income, sources of income and postcode. Parental stress was assessed using two items: Q1 *How would you rate your average level of stress in the past 30 days* and Q2 *How would you rate your ability to manage stress in the past 30 days?* Both items were measured on a scale of 1 (not stressed) to 10 (being very stressed) (Errisuriz, Pasch, and Perry 2016; Fulkerson et al. 2019; Nelson et al. 2008). A parental stress index was calculated by dividing the first parental stress item (Q1) by the second (Q2). It was then dichotomised, where an index < 1 indicated well‐managed stress and a score of ≥ 1 indicated unmanaged stress (Errisuriz, Pasch, and Perry 2016; Fulkerson et al. 2019; Nelson et al. 2008). Information on the child included their age, sex, breast‐ and bottle‐feeding practices and age when complementary foods were commenced.

### 2.3 Data Analysis

Data were exported from REDCap into the Statistical Package for Social Sciences (IBM SPSS version 29) program where it was cleaned for subsequent analyses. Multiple imputation (MI) was used for missing data using the 5% rule (Dahiru 2008). Income was converted to equivalised income, to account for household size. As income was a categorical variable, the midpoint of each income category was used. The equivalence factor was built by giving one point to the first adult, 0.5 to subsequent adults and children over 15 years old and 0.3 to each child under the age of 15 years and summing up all points. The final value of equivalised income was the midpoint of the range reported by the participant, divided by the equivalence factor (Australian Bureau of Statistics 2016).

All continuous variables were tested for normality. Preliminary analyses included non‐parametric independent samples tests to compare sociodemographic data between questionnaire completers and non‐completers and other independent groups; Spearman's correlations were used to investigate initial relationships between continuous variables, primarily to check for potential multicollinearity within predictor variables. Summary statistics were calculated for feeding practices, mealtime structure, family meal environment, household chaos, food security status and sociodemographic data. Cronbach's alpha was used to assess the internal consistency of the feeding practices, mealtime structure and family meal environment scores due to the study sample being different from the original sample the questionnaire was validated in. Cronbach's alpha values above 0.6 were considered acceptable (Jansen et al. 2014). Hierarchical regression was used to investigate associations between feeding practices, household chaos, household food insecurity and selected sociodemographic variables (parent age, educational level and number of children). These sociodemographic variables were selected a priori. Parent age and education are routinely included in feeding practice research (Arlinghaus and Laska 2021; Daniels et al. 2015; Hurley, Cross, and Hughes 2011; Jansen et al. 2021). The number of children was considered based on the hypothesis that having multiple children may make it challenging to feed responsively, especially in chaotic environments at risk of food insecurity. In the first model, the association between each feeding practice and sociodemographic variables was assessed. HC was added to the second model and HFI was added to the final model. Collinearity diagnostics (Variance Inflation Factor (VIF) and tolerance) were checked. No variables were excluded from the final models as no collinearity assumptions were violated.

### 2.4 Ethics Statement

RFiTT was approved by the Children's Health Queensland (reference number: LNR/21/QCHQ/72314) and the Queensland University of Technology (approval number: 2021000193) Human Research Ethics Committees.

## 3 Results

### 3.1 Participant Characteristics

The questionnaire was accessed by 601 participants, of which 290 consented. During data cleaning, it was observed that a significant number of participants had a child outside the age range of 6 months to 2 years. To maximise the data collected in the sample, the research team decided to extend the inclusion age from 5 months (on the basis that the child had commenced solids) to 35 months to accommodate children who were older than 2 years old but under the age of 3 years old (*n* = 11). Thirty‐five participants outside the extended age range were excluded. A further 42 participants were excluded because they only provided sociodemographic data and did not complete sections of the questionnaire that were related to outcomes of interest. There were no statistically significant differences between the sociodemographic characteristics of questionnaire completers versus non‐completers. After excluding non‐completers, 213 participants were available for analyses (Figure 1).

Table 1 describes the sociodemographic characteristics of parents in the sample. Nearly all parents were mothers (97%) with a median age of 31 years (IQR: 28–36). Forty‐four percent were in the lowest two quintiles of equivalised income. There was only one child in 50% of households. The median age of the index child was 12 months (IQR 8–17) and 50% were boys. Approximately 47% of the children were currently breastfed, while a further 43% were breastfed but had stopped at the time of the survey. Over 75% of parents indicated that they were either entirely responsible or mostly responsible for planning and preparing meals for their child and feeding or assisting their child with eating.

**Table 1:** Characteristics of parents and their household (*n* = 213).

	Median (IQR), or *n* (%)
Age (years)	31 (28,36)
Born in Australia	166 (78)
Ethnicity	
Australian	150 (70)
Australian Aboriginal and or Torres Strait Islander	2 (1)
Other	61 (29)
Highest level of education	
Tertiary	87 (59)
High school/diploma/certificate	124 (41)
Relationship status	
Partnered^a^	187 (88)
Not partnered	25 (12)
Relationship to child	
Parent	207 (97)
Father	2 (1)
Other	4 (2)
No. of children	
1 child	106 (50)
> 1 child	105 (50)
No. of adults in the household	
1	26 (12)
2	163 (77)
3 or more	24 (11)
No. of times families have moved homes in the last 12 months	
0	147 (69)
1	51 (24)
> 2	15 (7)
Body Mass Index^b^	
Underweight (< 18.5 kg/m^2^)	4 (2)
Healthy weight (18.5–24.99 kg/m^2^)	54 (25)
Overweight (> 25–29.99 kg/m^2^)	68 (32)
Obese (> 30 kg/m^2^)	89 (41)
Equivalised Income AUD per annum^c^	
0–18571	46 (21)
18572–32498	49 (23)
32499–40975	29 (14)
40976–45258	40 (19)
45259	40 (19)
Prefer to not say	9 (4)
Source of Income	
Salary and wages	145 (68)
Government support, pension, or assistance	59 (28)
Other	8 (4)
Prefer not to say	1 (1)
SEIFA IRSD quintile^d^	
1	47 (23)
2	36 (18)
3	42 (20)
4	37 (18)
5	44 (21)
Parental stress index	
Well‐managed stress	45 (27)
Unmanaged stress	119 (73)
Household CHAOS (HC)	4 (2,7)
Household food insecurity (HFI)	
Food secure	42 (24)
Food secure	19 (45)
Marginally food secure	23 (55)
Food insecure	135 (76)
Low food security (with hunger, moderate)	63 (47)
Very low food security	70 (53)

^a^ Partnered was defined as married, de facto and together but living apart. Not partnered was defined as single, divorced and widowed.

^b^ Body Mass Index categorised using World Health Organisation Cut‐offs.

^c^ Equivalised Total Household Income is total household income adjusted with an equivalence scale to enable comparison of income levels between households of differing size and composition.

^d^ Postcode used to derive Index of Relative socioeconomic Disadvantage (IRSED), where quintile 1 is the most disadvantaged through to quintile 5 being least disadvantaged.

Seventy‐six percent of families were food insecure and 73% of parents reported unmanaged stress in the last 30 days. HC was relatively low with a median of 4.0 (IQR: 2.0–7.0). HC was lower in families with one child (median of 4.0, IQR 1.0–6.0) compared to households with multiple children (median of 5.9, IQR 3.0–9.0) and this difference was statistically significant (*p* = 0.004).

HFI was associated with equivalised income (*ρ* = −0.313, *p* < 0.001), parental stress index (*ρ* = 0.295, *p* < 0.001) and HC (*ρ* = 0.169, *p* = 0.033). HC was associated with the parental stress index (*ρ* = 0.265, *p* < 0.001) but not income. Parental stress index was not associated with any of the feeding practices, mealtime structure or the feeding environment.

### 3.2 Associations Between Feeding Environment, Mealtime Structure and Practices, Household Chaos and HFI

All feeding practices, mealtime structure and mealtime environment demonstrated acceptable internal consistency (Table 2). ‘feeding on demand’, a measure of mealtime structure, had a median score of 3.5 (IQR: 3.0–4.0) (Table 2). Both HC and HFI were negatively associated with ‘feeding on demand’ (Table 3). Using ‘food to calm’ had a median score of 2.0 (IQR 1.5–2.5). HC explained 5.8% of the variation in using ‘food to calm’; however, HFI was not associated with this practice. Persuasive feeding had a median score of 2.3 (IQR: 1.7–3.0). While persuasive feeding was weakly correlated with child age in preliminary bivariate analyses (*ρ* = 0.208, *p* < 0.011), it was not associated with child age after adjusting for parent age, education level and number of children. Persuasive feeding was not associated with HC or HFI.

**Table 2:** Median scores of feeding practices, mealtime structure and mealtime environment of parents living with financial hardship.

Feeding practice	Number of items	Cronbach's alpha	Median^a^ (IQR)
Family meal environment	4	0.80	4.0 (3.5–4.8)
Feeding on demand	4	0.67	3.5 (3.0–4.0)
Food to calm	6	0.77	2.0 (1.5–2.5)
Parent‐led feeding	4	0.77	1.8 (1.0–2.5)
Persuasive feeding	7	0.84	2.3 (1.7–3.0)
(Non)‐food as reward	9	0.92	1.33 (1.0–2.0)

^a^ Scores range from 1 to 5.

**Table 3:** The association between feeding practices, household chaos and household food insecurity.

	Model 1	Model 2	Model 3
**Feeding on demand**			
Standardised coefficients			
Parent age (years)	0.178*	0.173*	0.119
Tertiary (ref) versus non‐tertiary education	0.038	0.030	−0.006
One (ref) versus multiple children	−0.055	−0.112	−0.116
Household chaos		−0.204*	−0.159
Household food insecurity			−0.216**
*R* ^2^ change	0.040	0.038*	0.040*
*F*_(5,147)_	2.092	3.164*	3.974**
**Food to calm**			
Standardised coefficients			
Parent age (years)	0.187*	0.193*	0.198
Tertiary (ref) versus non‐tertiary education	0.076	0.086	0.090
One (ref) versus multiple children	−0.076	−0.006	−0.005
Household chaos		0.252**	0.247**
Household food insecurity			0.023
*R* ^2^ change	0.052*	0.058**	0.000
*F*_(5,147)_	2.740*	4.607**	3.678**
**Persuasive feeding**			
Standardised coefficients			
Parent age (years)	0.181*	0.185*	0.181*
Tertiary (ref) versus non‐tertiary education	−0.061	−0.056	−0.058
One (ref) versus multiple children	−0.011	0.029	0.029
Household chaos		0.144	0.147
Household food insecurity			−0.015
*R* ^2^ change	0.037	0.019	0.000
*F*_(5,147)_	1.913	2.199	1.754

* *p* < 0.05

** *p* < 0.01.

The predictive analysis could not be carried out for ‘parent‐led feeding’, using ‘(non)‐food rewards’ or ‘family meal environment’, due to the parent responses being severely skewed. For ‘parent‐led feeding’, the median score was 1.8 (IQR (1.0–2.5), with almost 88% of the sample indicating never or rarely using this practice. Using ‘(non)‐food as reward’ had a median score of 1.33 (IQR 1.0–2.0) and 94% of the sample reported never or rarely using this practice. Bivariate analysis (see supplementary files) indicated it was associated with child age (*ρ* = 0. 0.385, *p* < 0.001) and household chaos (*ρ* = 0.259, *p* = 0.016). The median score for ‘family meal environment’ was 4.0 (IQR 3.5–4.8), with 80% of parents indicating often or always using this practice. It was also associated with child age in bivariate analysis (*ρ* = 0.258, *p* = 0.001).

## 4 Discussion

Parental feeding practices are an important precursor for the quality of a child's relationship with food and their growth and development (Black and Aboud 2011). Previous research mainly focused on high‐income families. This study is the first to investigate the family meal environment, mealtime structure and nonresponsive feeding practices among Australian parents with financial hardship. Results showed that three quarters of families faced food insecurity (HFI). Although the meal environment generally supported responsive feeding and coercive practices were low, higher household chaos (HC) and food insecurity were linked to less structured mealtimes. HC notably influenced practices such as using food to calm or reward more than HFI.

In a predominantly food‐insecure cohort with low levels of household chaos, it was promising to see the family meal environment was conducive to responsive feeding. Most parents indicated their child often or always ate with other family members and were given the same foods with age‐appropriate texture as the rest of the family. Parents indicated that regardless of whether the child was eating, they sat with the family during mealtimes. Parents indicated they often or always ate something while their child ate. Given the age of the children in our sample, it is unlikely that children would be left unattended during meals. Additionally, the COVID‐19 pandemic, which led to one or both parents being at home due to government lockdowns and a subsequent increase in flexible work‐from‐home arrangements, may have influenced these results (Adams et al. 2020; Beck and Hensher 2020, 2022; Cornell et al. 2022). This finding was consistent with other research involving food insecure families that reported eating family meals together was valued and an important point of connection (Baxter, Nambiar, et al. 2024; Hevesi, Downey, and Harvey 2024). However, it is important to note that while eating meals together as a family was valued, actually doing so may be impacted by age and number of children, the degree of food insecurity and the level of chaos in the household (Fiese et al. 2016; Marsh, Dobson, and Maddison 2020).

In this sample, the median score for ‘feeding on demand’ indicated a slightly more structured feeding environment. This score was comparable across several studies, including So and colleagues' study with low‐income fathers (So et al. 2024); a sample of high‐income parents who participated in the original validation of the FPSQ (Jansen et al. 2014) and another recent study examining the impact of COVID‐19 on structure‐related food parenting practices (Jansen et al. 2022). Among very young children, such as those in this sample, ‘feeding on demand’ could be seen as a beneficial practice, as children are fed according to their hunger cues rather than at fixed times. It remains unclear, however, whether ‘feeding on demand’ becomes less beneficial in older children, who may require more structure, rather than a ‘grazing’ pattern of eating (Jansen et al. 2021; Vaughn et al. 2015). ‘Feeding on demand’ was associated with both HFI and household chaos, which, explained 8% of the variation in the ‘feeding‐on‐demand’ score. As HFI and HC increased, the feeding‐on‐demand score decreased, indicating that dysfunction, coupled with HFI, resulted in less structured practices which is consistent with previous research (Baxter, Nambiar, et al. 2024; Fiese et al. 2016). It is positive to see that both chaos and HFI only explained 8% of the variation in the ‘feeding‐on‐demand’ score, demonstrating that families may be able to adapt to their circumstances and respond to their child in line with age and developmental stage.

The overall use of coercive, nonresponsive practices was low in this sample, possibly due to the children in the sample being quite young. This theory was supported by the findings of So et al. (2024) and Jansen et al. (2014), where higher scores for these practices were observed among children who were, on average, 12 months older than this study sample (Jansen et al. 2014; So et al. 2024). Environments that are prone to noise, dysfunction and instability may make it challenging for parents to feed children responsively (Baxter, Nambiar, et al. 2024; Marsh, Dobson, and Maddison 2020). This was evident, to a degree, in this study. Preliminary bivariate analyses indicated a weak but statistically significant positive association between HC and using ‘(non)‐food rewards’. However, the data were too skewed for further predictive analyses. Using ‘food to calm’ was also positively associated with HC, although HC only explained 6% of the variation in the ‘food to calm’ score, likely due to the overall level of chaos being low. As half the families in this study consisted of two adults and one very young child, it is possible that not all questions in the HC scale were applicable to our sample, particularly those related to noise levels. This study demonstrated that households with multiple children had a significantly higher HC score than households with one child suggesting the level of chaos may increase as family size increases and children become older (Fulkerson et al. 2019). Our findings were consistent with other recent Australian studies that found associations between certain nonresponsive feeding practices and HC (Baxter, Nambiar, et al. 2024; So et al. 2024).

Overall, HFI was not associated with any of the nonresponsive feeding practices characterised in this study. This contrasted with So and colleagues' (2024) study of food‐insecure fathers, where HFI was associated with ‘persuasive feeding’ and ‘parent‐led feeding’. The children in So's study were the same age as the children in this study. Orr et al. (2020) also found that food‐insecure parents of 2‐year olds used pressuring feeding behaviours (Orr et al. 2020). This contrast in findings may be due to the sample being predominantly food insecure and the lack of variability in the sample meant this relationship was not apparent. However, a more optimistic reason may be that food‐insecure families are resilient and able to demonstrate the capacity to use responsive feeding practices despite nonoptimal environments. Parents living with disadvantage and financial hardship have demonstrated good understanding when it comes to recognising hunger and satiety cues, understanding the importance of providing food variety and modelling healthy eating behaviours regardless of their living situation (Agrawal et al. 2018; Almaatani et al. 2023; Baxter et al. 2022; Hevesi, Downey, and Harvey 2024; Johnson et al. 2011).

While not screening specifically for food‐insecure families, the prevalence of food insecurity in this study was high at 76% (low and very low food security). A further 13% of the sample were marginally food secure, indicating a level of anxiety about feeding household members. The prevalence reported in this study was comparable to two recent Australian studies that used the same HFSSM questions. The first, among participants with entrenched hardship and disadvantage, found 83% were food insecure and a further 8% were marginally secure (Seivwright, Callis, and Flatau 2020). The second study, among a cohort of fathers living with financial difficulties, found 77% were food insecure with a further 14% marginally food secure (So et al. 2024). The pandemic caused food shortages in Australian supermarkets due to supply chain disruptions, panic buying and lockdown measures, although this varied across states and had largely resolved at the time this survey was conducted. Therefore, it remains unclear how these changes may have directly impacted feeding practices in the Australian context in the aftermath of the pandemic. Jansen et al. (2021) found that in an American cohort of children aged 2–12 years, higher COVID‐19‐specific stress was associated with more emotional and instrumental feeding, but this was coupled with more structure and engaged mealtimes (Jansen et al. 2021). A review by Luo et al. (2022) also found that during the pandemic (2020–2021), parents of children aged 3–18 years used high levels of coercive control and reduced rules and limits (Luo et al. 2022).

Over three quarters of parents (nearly all mothers) in our study indicated that they were either entirely responsible or primarily responsible for planning and preparing meals for their child and feeding/assisting their child with eating. In a food‐insecure environment, this is a challenging responsibility, where one parent bears the burden of food work. This substantially increases the mental load parents must carry (Agrawal et al. 2018; Baxter et al. 2022). It is not surprising that 73% of parents also reported unmanaged stress in the last 30 days. The combination of HFI, primary responsibility for food work and several other factors such as moving homes more than once in the last 12 months may have contributed to prolonged stress and anxiety in this sample. Parental stress was not associated with the family meal environment, mealtime structure or any of the nonresponsive feeding practices characterised in this study. This finding was consistent with a recent study in children less than 5 years of age (Almaatani et al. 2023); however, another US study with children over the age of 5 years indicated that parental stress was associated with restrictive feeding practices in food‐insecure households (Berge et al. 2020, 2017). These mixed findings may suggest that parents of older children may be more likely to use nonresponsive practices in certain stressful situations (Adams et al. 2020).

This study has several limitations that may impact the generalisability of the results. First, the data were collected during the latter period of the COVID‐19 pandemic. The pandemic resulted in significant changes to the household structure, with one or both parents spending more time at home during government‐mandated restrictions to reduce the spread of COVID‐19. It remains unclear whether parents in this study continued to work from home or returned to pre‐COVID work arrangements after the restrictions were lifted. This meant that there were potentially more opportunities to have meals together and more feeding interactions across the day if parents continued to work from home. Second, the prevalence of HFI in this sample was exceptionally high. We used financial hardship as a proxy for HFI. However, the pandemic resulted in job losses and changes to income, and coupled with the rising cost of living, which would have also contributed to the higher HFI prevalence. Third, the study sample lacked variability with limited cultural diversity, the majority were food insecure and the children were very young and within a narrow age range. Responses to some practices within the sample were highly skewed. As a result, predictive analysis could not be carried out for some practices, so it remains unclear whether either household chaos and HFI had an impact on these feeding practices and the environment. Lastly, it would have been desirable to capture more practices around structure and autonomy support among food‐insecure families. Findings from qualitative studies have suggested that some practices such as role‐modelling and providing adequate food variety may be challenging for food‐insecure households (Arlinghaus and Laska 2021; Baxter, Nambiar, et al. 2024; Baxter et al. 2022). Understanding this among a larger cohort would enable future interventions to develop suitable strategies to support families in these areas.

This study has several strengths. First, our targeted recruitment strategies effectively reached families experiencing financial hardship, particularly those underrepresented in feeding practice research. This enabled the feeding environment, mealtime structure and nonresponsive practices to be characterised, some of which were associated with HC and HFI. Future interventions can prioritise addressing these practices by offering strategies tailored to food‐insecure families. Second, despite data collection occurring during the latter part of the COVID‐19 pandemic, it provided valuable insights into feeding practices during a stressful period. These findings demonstrate that parents can remain resilient and continue using responsive feeding practices despite disruptions. Finally, the results will benefit maternal and child health nurses (MCHNs) and other health professionals supporting child feeding. MCHNs, who have substantial contact with parents during the early years, can guide responsive feeding practices and minimise coercive practices explored in this study.

Despite financial hardship and food insecurity, the level of household chaos was low, the family meal environment was conducive to responsive feeding and the use of nonresponsive feeding practices was low. Increasing chaos and food insecurity severity resulted in less structured mealtimes, while household chaos had a greater impact on the use of certain nonresponsive feeding practices than HFI. Further investigations are needed to fully understand the relationship between HFI, HC and feeding practices. Future studies on child feeding would benefit from recruiting a more equal distribution of food secure and insecure families with a wider age range of children, as the use of nonresponsive feeding practices was more apparent in older children in other studies. Child feeding interventions should consider how household chaos and food insecurity can impact parents' ability to engage in responsive feeding practices. Codesign with end users using a strengths‐based approach may be useful in designing such interventions.

## Author Contributions

S.N. was responsible for funding acquisition, performed data cleaning and analyses, wrote the final version of the manuscript and was the primary supervisor of L.S. and L.M. for their Bachelor of Nutrition and Dietetics Honours dissertation. L.M. and L.S. jointly assisted in data cleaning, contributed to the data analysis and wrote the first draft of the manuscript. R.A.B. and D.G. were responsible for funding acquisition and contributed to the writing and review of the manuscript. R.A.P. was responsible for writing and reviewing the manuscript. K.A.B. was responsible for data collection, contributing to writing and reviewing the manuscript and co‐supervising L.M. and L.S.

## Conflicts of Interest

The authors declare no conflicts of interest.

## Supporting information

Supporting information.

## Data Availability

The data that support the findings of this study are available on request from the corresponding author. The data are not publicly available due to privacy or ethical restrictions.
