# Supplementary material for: Feeding Practices Used by Australian Parents of Young Children Living With Food Insecurity and Household Chaos
Source: Matern Child Nutr. 2024 Nov 25;21(2):e13770. doi: 10.1111/mcn.13770 (PMC11956056; doi:10.1111/mcn.13770)
Supplement: Supplementary file 1 — Supporting information. [file MCN-21-e13770-s001.pdf]

Feeding Practices used by Australian parents of young children living with food insecurity and household chaos.
Supplementary File 1: Preliminary bivariate analyses

	Spearman's rho	Parent age in years	child age in months	Household Chaos Score	USDA FS Score	Parental stress index	Equivalised Income	Feeding Demand on Score	Food to calm score	Persuasive feeding score	Parent-led Feeding Score	Using food as reward score	Family Meal Environment Score
Feeding on Demand Score	Correlation Coefficient	.202**	0.063	-0.127	-.255**	-0.131	-0.008		-0.016	.186*	.284**	0.117	0.070
	Sig. (2-tailed)	0.007	0.445	0.119	0.001	0.104	0.914		0.829	0.012	0.000	0.139	0.353
	N	181	150	153	167	156	174		181	181	180	162	180
Food to calm score	Correlation Coefficient	.146*	0.124	.217**	0.026	0.037	-0.030	-0.016		.366**	.262**	.325**	-0.022
	Sig. (2-tailed)	0.049	0.129	0.007	0.739	0.646	0.692	0.829		0.000	0.000	0.000	0.765
	N	181	150	153	167	156	174	181		181	180	162	180
Persuasive feeding score	Correlation Coefficient	.175*	.208*	.186*	-0.044	-0.015	0.023	.186*	.366**		.536**	.532**	-0.119
	Sig. (2-tailed)	0.019	0.011	0.021	0.574	0.857	0.764	0.012	0.000		0.000	0.000	0.113
	N	181	150	153	167	156	174	181	181		180	162	180
Parent-led Feeding Score	Correlation Coefficient	.151*	0.008	0.067	-0.054	0.007	0.055	.284**	.262**	.536**		.445**	-0.109
	Sig. (2-tailed)	0.043	0.923	0.413	0.485	0.927	0.473	0.000	0.000	0.000		0.000	0.144
	N	180	150	153	167	156	173	180	180	180		162	180
Using food as reward score	Correlation Coefficient	0.124	.385**	.175*	0.145	0.071	-.264**	0.117	.325**	.532**	.445**		-0.017
	Sig. (2-tailed)	0.116	0.000	0.040	0.077	0.400	0.001	0.139	0.000	0.000	0.000		0.831
	N	162	136	139	150	143	157	162	162	162	162		162
Family Meal Environment Score	Correlation Coefficient	0.018	.258**	0.014	0.058	0.086	-0.022	0.070	-0.022	-0.119	-0.109	-0.017	
	Sig. (2-tailed)	0.813	0.001	0.868	0.460	0.288	0.774	0.353	0.765	0.113	0.144	0.831	
	N	180	150	153	167	156	173	180	180	180	180	162	
Parent age in years	Correlation Coefficient		.263**	-0.012	-.294**	-0.073	0.106	.202**	.146*	.175*	.151*	0.124	0.018
	Sig. (2-tailed)		0.001	0.881	0.000	0.355	0.132	0.007	0.049	0.019	0.043	0.116	0.813
	N		157	160	175	164	204	181	181	181	180	162	180
child age in months	Correlation Coefficient	.263**		0.139	-0.046	0.055	-0.062	0.063	0.124	.208*	0.008	.385**	.258**
	Sig. (2-tailed)	0.001		0.082	0.568	0.492	0.448	0.445	0.129	0.011	0.923	0.000	0.001
	N	157		157	157	156	151	150	150	150	150	136	150

Feeding Practices used by Australian parents of young children living with food insecurity and household chaos.
Supplementary File 1: Preliminary bivariate analyses

	Spearman's rho	Parent age in years	child age in months	Household Chaos Score	USDA FS Score	Parental stress index	Equivalised Income	Feeding Demand on Score	Food to calm score	Persuasive feeding score	Parent-led Feeding Score	Using food as reward score	Family Meal Environment Score
Household Chaos Score	Correlation Coefficient	-0.012	0.139		.169*	.344**	-0.053	-0.127	.217**	.186*	0.067	.175*	0.014
	Sig. (2-tailed)	0.881	0.082		0.033	0.000	0.511	0.119	0.007	0.021	0.413	0.040	0.868
	N	160	157		160	159	154	153	153	153	153	139	153
USDA FS Score	Correlation Coefficient	-.294**	-0.046	.169*		.295**	-.313**	-.255**	0.026	-0.044	-0.054	0.145	0.058
	Sig. (2-tailed)	0.000	0.568	0.033		0.000	0.000	0.001	0.739	0.574	0.485	0.077	0.460
	N	175	157	160		164	169	167	167	167	167	150	167
Parental stress index	Correlation Coefficient	-0.073	0.055	.344**	.295**		-0.147	-0.131	0.037	-0.015	0.007	0.071	0.086
	Sig. (2-tailed)	0.355	0.492	0.000	0.000		0.066	0.104	0.646	0.857	0.927	0.400	0.288
	N	164	156	159	164		158	156	156	156	156	143	156
Equilvilised Income	Correlation Coefficient	0.106	-0.062	-0.053	-.313**	-0.147		-0.008	-0.030	0.023	0.055	-.264**	-0.022
	Sig. (2-tailed)	0.132	0.448	0.511	0.000	0.066		0.914	0.692	0.764	0.473	0.001	0.774
	N	204	151	154	169	158		174	174	174	173	157	173

*. Correlation is significant at the 0.05 level (2-tailed).

**. Correlation is significant at the 0.01 level (2-tailed).
